# Supplementary figures and images for: Physiological response mechanism of Machilus faberi Hemsl under drought stress and rewatering
Source: PeerJ. 2025 Sep 10;13:e19855. doi: 10.7717/peerj.19855 (PMC12433196; doi:10.7717/peerj.19855)

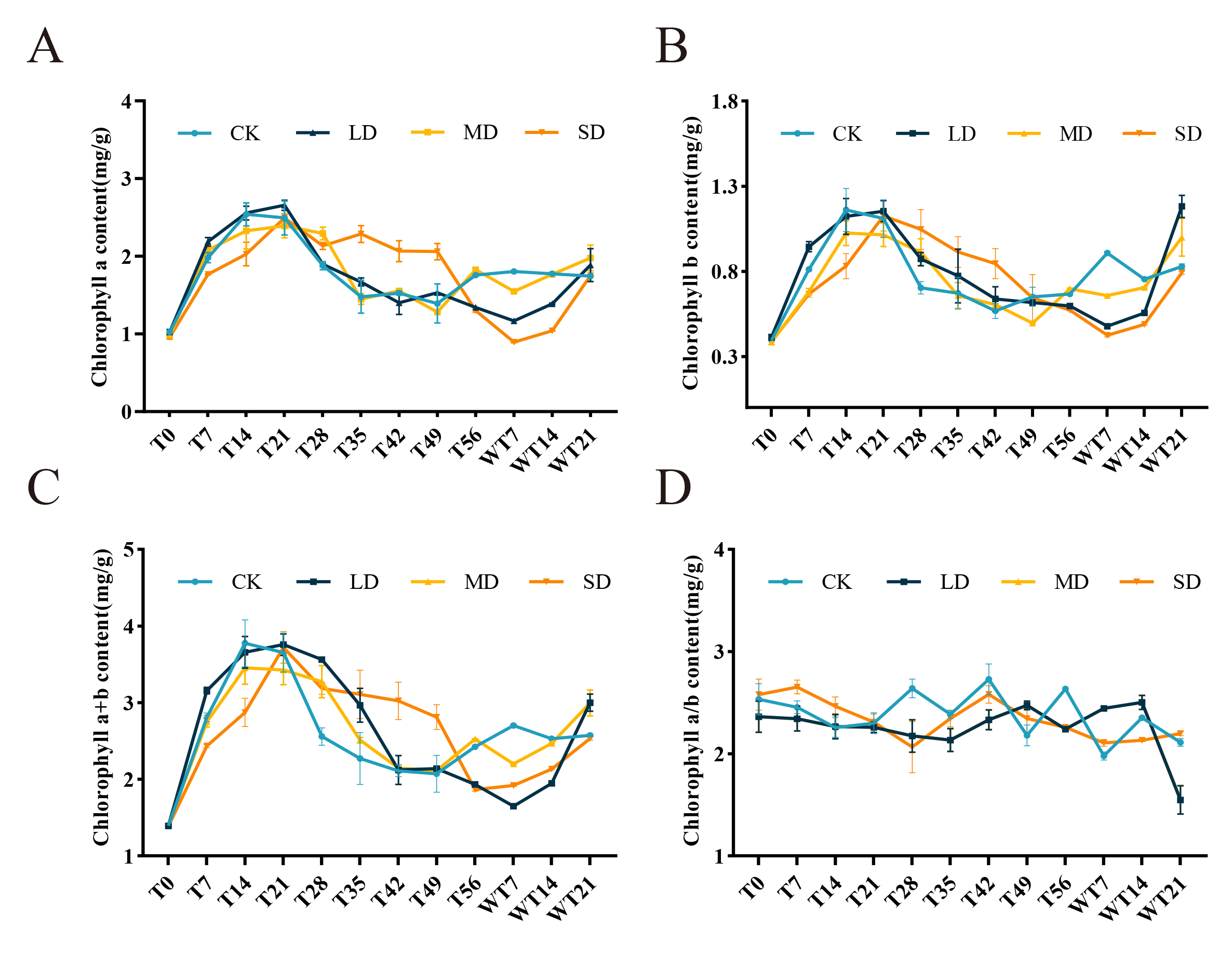

Supplement: Supplemental Information 2 — (A) Chl a; (B) Chl b; (C) Chl a/b; (D) Chl a+b. [file peerj-13-19855-s002.jpg]

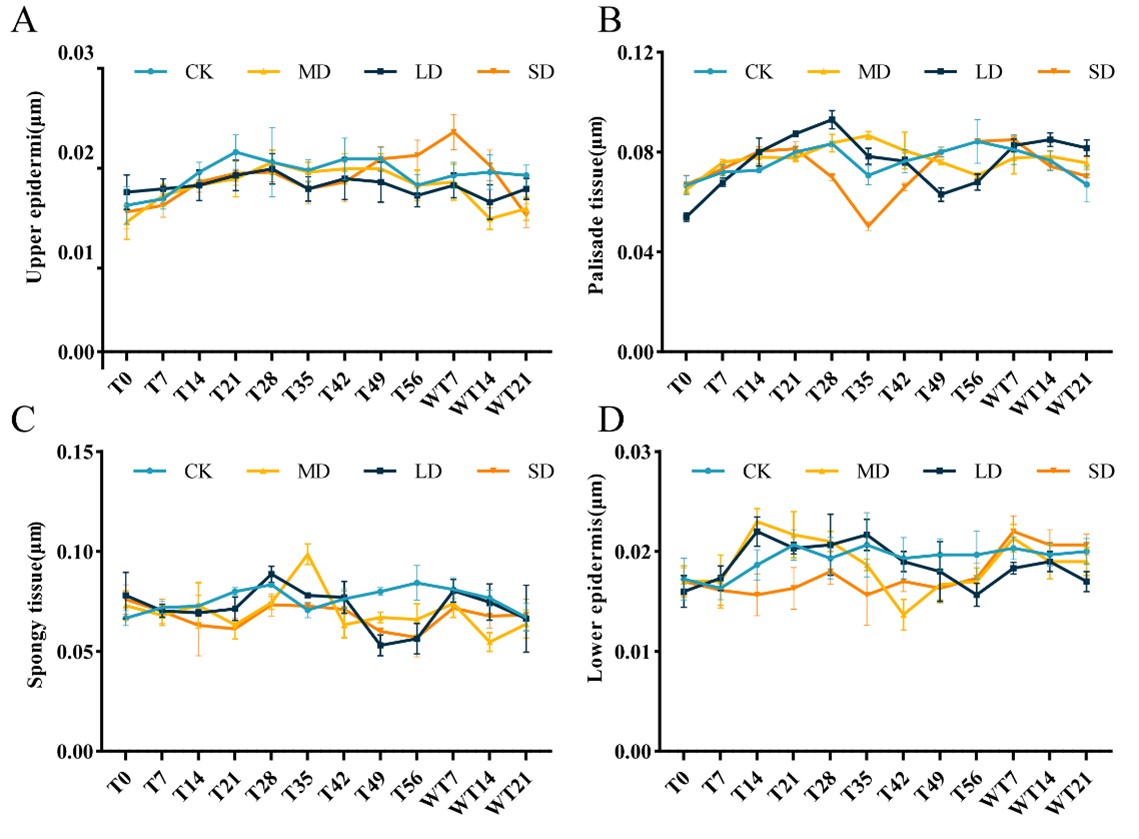

Supplement: Supplemental Information 3 — (A) Upper epicuticle; (B) Palisade tissue; (C) Spongy tissue; (D) Lower epidermis. [file peerj-13-19855-s003.jpg]

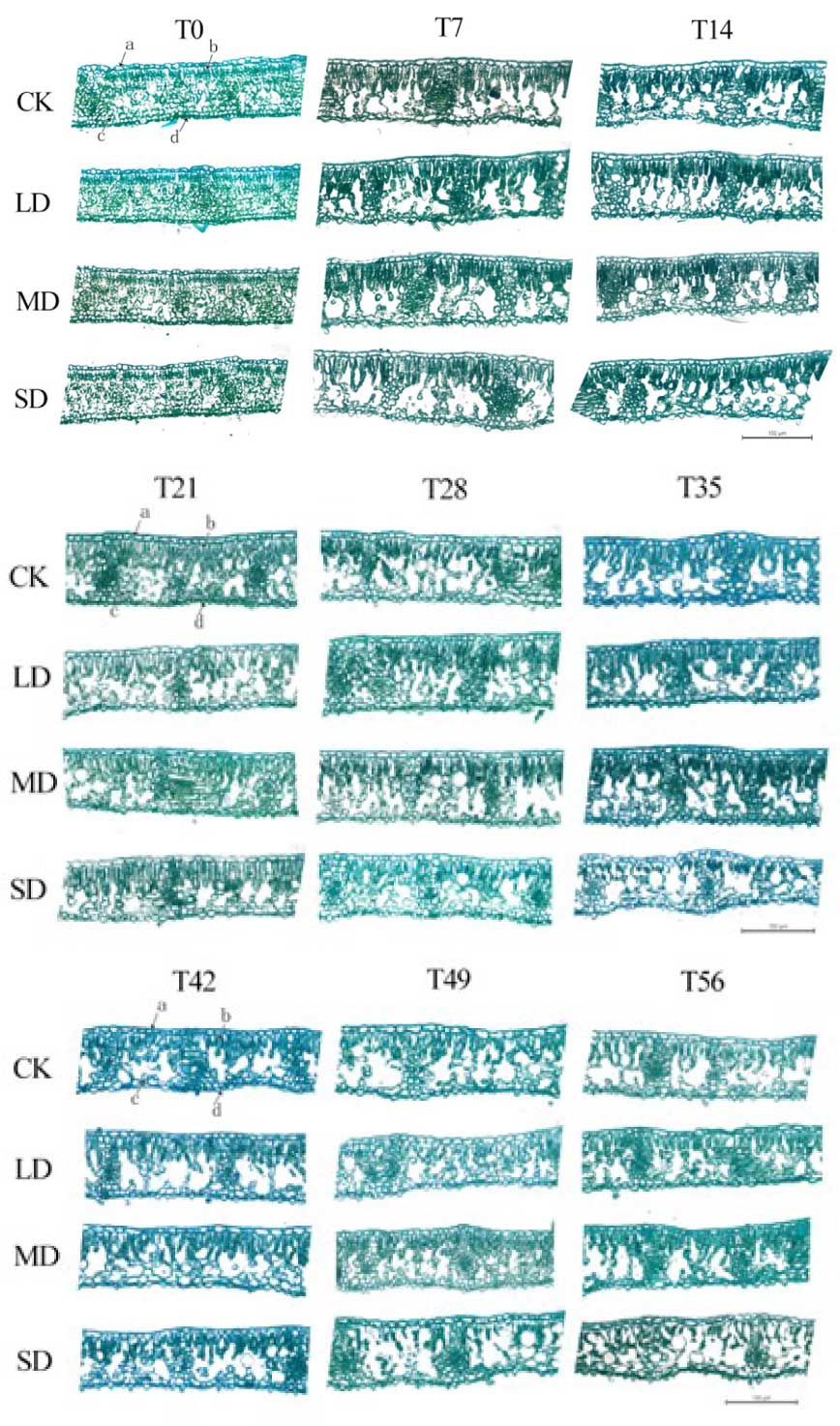

Supplement: Supplemental Information 4 — a is the epidermis; b is the palisade tissue; c is sponge tissue; d is the Hypodermis; Scale 100 µ. [file peerj-13-19855-s004.jpg]

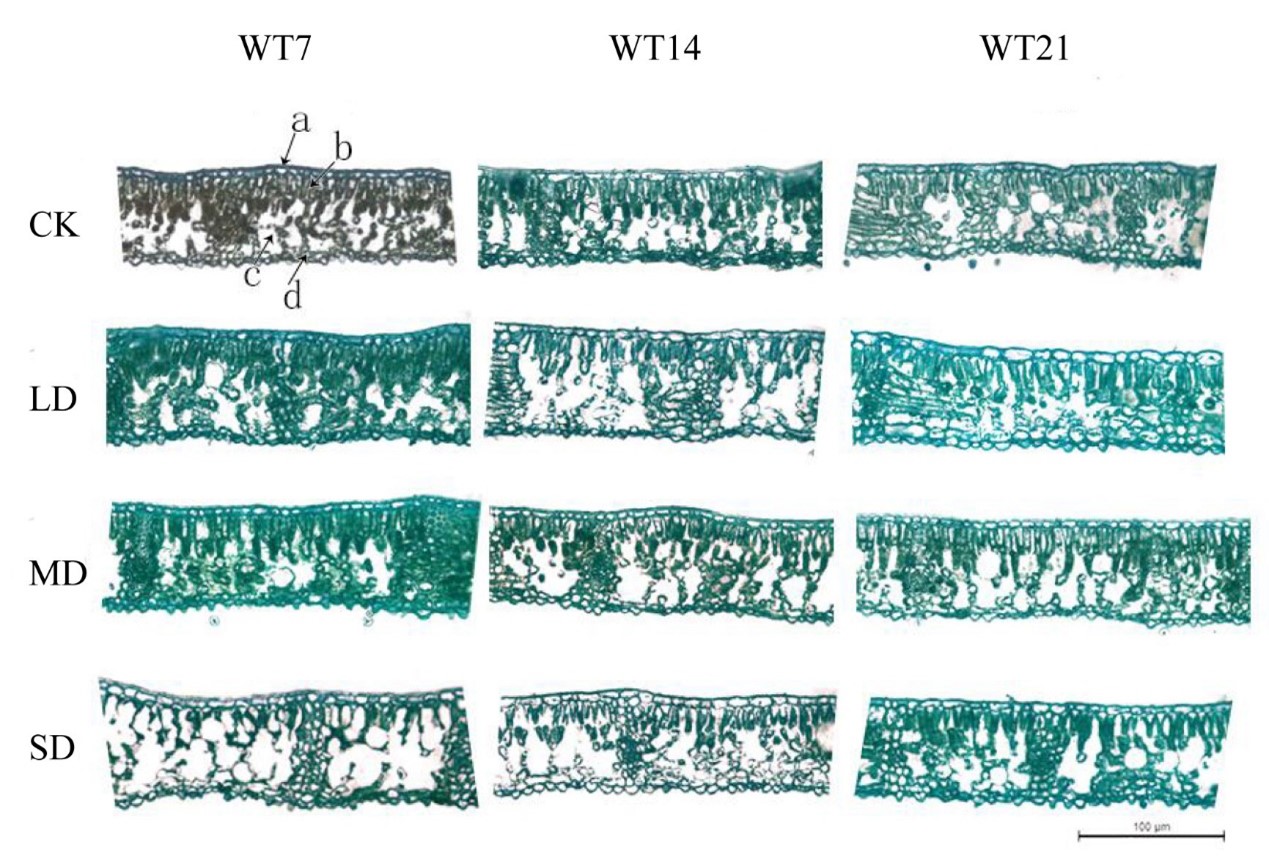

Supplement: Supplemental Information 5 — a is the epidermis; b is the palisade tissue; c is sponge tissue; d is the Hypodermis; Scale 100 µ. [file peerj-13-19855-s005.jpg]

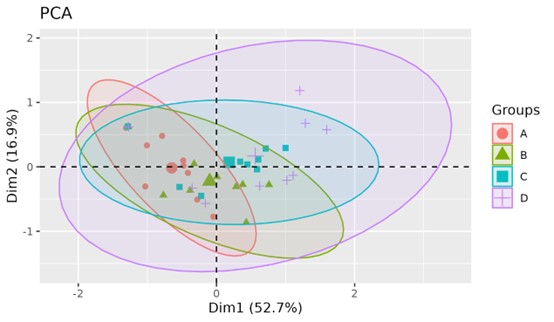

Supplement: Supplemental Information 6 — (A) CK; (B) LD; (C) MD; (D) SD. [file peerj-13-19855-s006.jpg]
